# Synthesis of Oligodeoxynucleotide Containing Pseudo‐Deoxycytidine and Its Triphosphate Derivative

**DOI:** 10.1002/cpz1.70101

**Published:** 2025-03-17

**Authors:** Ryo Miyahara, Yosuke Taniguchi

**Affiliations:** ^1^ Graduate School of Pharmaceutical Sciences Kyushu University Fukuoka Japan; ^2^ Faculty of Medicine, Dentistry and Pharmaceutical Sciences Okayama University Okayama Japan

**Keywords:** artificial nucleic acid, 2‐hydroxy‐2'‐deoxyadenosine, 2‐OH‐dA, pseudo‐dC, pseudo‐deoxycytidine, tautomeric structure, unnatural base pair

## Abstract

This article describes a detailed synthetic protocol for the preparation of oligodeoxynucleotide (ODN) containing pseudo‐deoxycytidine (ψdC) and its triphosphate derivative (ψdCTP). These molecules were synthesized as novel compounds that recognize iso‐2'‐deoxyguanosine (iso‐dG) in DNA. Iso‐dG is one of the tautomers of 2‐hydroxy‐2'‐deoxyadenosine (2‐OH‐dA), which is known as an oxidatively damaged nucleobase, and its selective recognition in DNA is expected to play a very important role in the diagnosis and pathogenesis of diseases. The hydroxyl groups of the known glycal compound were protected with silyl groups, and then coupled with 5‐iodouracil under Mizorogi‐Heck reaction conditions, yielding ψdU after desilylation and diastereoselective reduction. The endocyclic amino group of ψdU was protected by the benzyl group. Subsequently, the carbonyl group at the 6‐position of the nucleobase was activated and converted to an amino group through treatment with aqueous ammonia. The benzyl group was removed, and the exocyclic amino group was protected with a benzoyl group. On one hand, the silyl groups at the 3’ and 5’ positions were deprotected, converted into a phosphoramidite unit, and incorporated into an ODN. On the other hand, the hydroxyl group at the 5’ position was selectively deprotected and then directly converted into the triphosphate using Van Boom's reagent under acidic conditions. © 2025 The Author(s). Current Protocols published by Wiley Periodicals LLC.

**Basic Protocol 1**: Synthesis of ODNs having ψdC and ψdCTP

**Basic Protocol 2**: Melting temperature of duplex formation between ODNs containing ψdC unit and 2‐OH‐dA

**Basic Protocol 3**: A single nucleotide primer extension reaction of ψdCTP for a template strand containing 2‐OH‐dA

## INTRODUCTION

**Figure 1:**
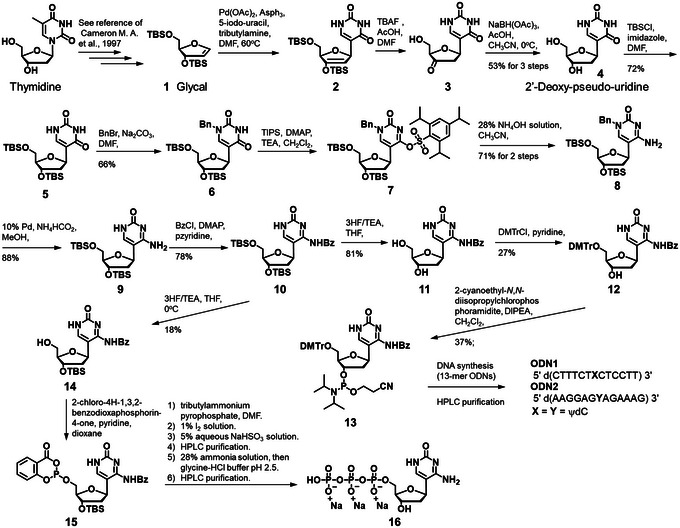
Synthetic pathway from glycal to ODNs having ψdC and ψdCTP.

**Figure 2:**
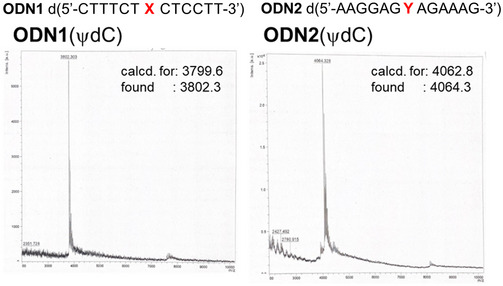
MALDI‐TOF MS (negative mode) chart of ODNs containing ψdC.

**Figure 3:**
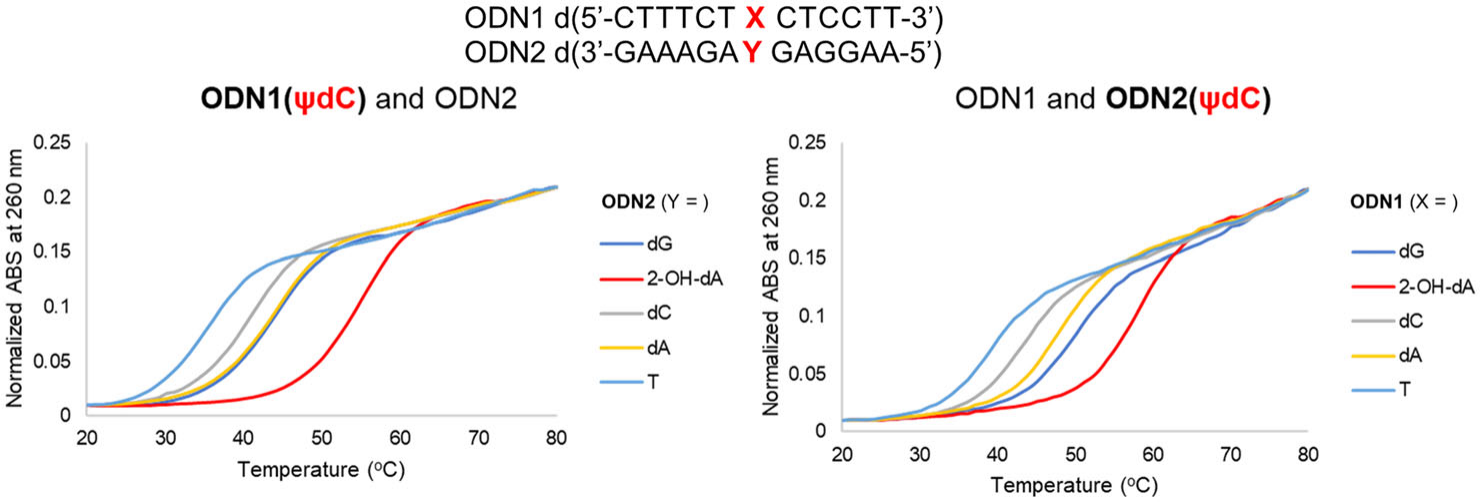
Melting temperature of duplex formation between ODN1 and ODN2. Conditions: 3 µM of each ODN in the buffer containing 100 mM NaCl, 7.5 mM MgCl_2_, and 5 mM sodium phosphate at pH 6.9. The absorbance at 260 nm was measured as the temperature was increased by 1°C/min from 20° to 80°C.

**Figure 4:**
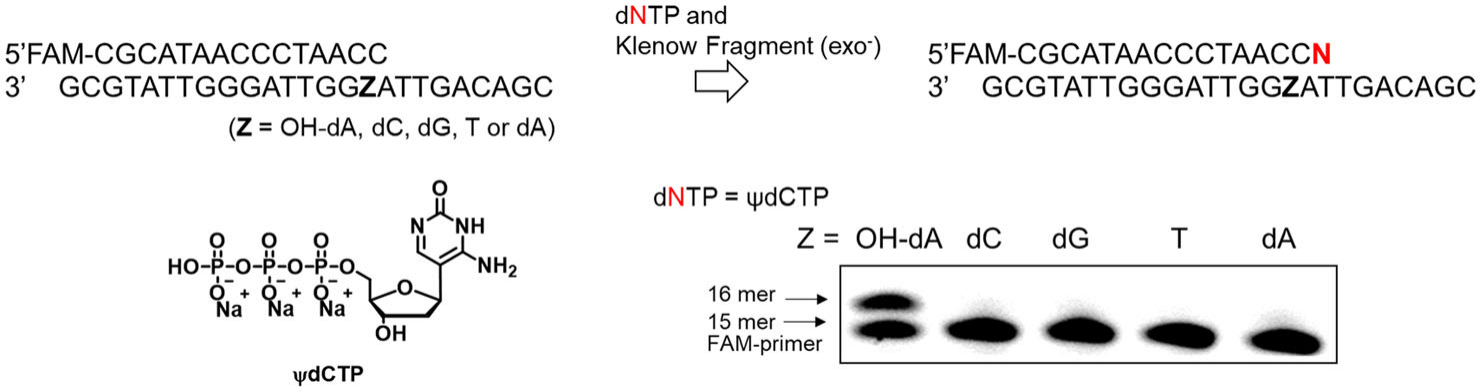
Single nucleotide primer extension reaction of ψdCTP for the template (Z) (Z = 2‐OH‐dA, dC, dG, T, and dA). Conditions: (**A**) 1.0 µM of the 15‐mer/25‐mer FAM‐labeled primer‐template (Z) duplex, 0.1 unit/µl Klenow Fragment (exo^–^), 10 mM Tris·HCl (pH 7.9), 50 mM NaCl, 10 mM MgCl_2_, 1 mM DTT, and 25 µM dNTPs incubated for 1 min in a reaction volume of 10 µl.

The standard features of DNA are essential for maintaining biological function. However, DNA undergoes continuous damage due to external sources, such as radiation, ultraviolet light, and chemicals, as well as internal sources, including reactive oxygen species generated during metabolism. Among the many types of DNA damage, oxidative modifications in the nucleobases can lead to non‐complementary base pairing, resulting in transversion mutations (Cooke et al., 2003; Kamiya & Kasai, 1997; Suzuki & Kamiya, 2016). While most damage occurs randomly, mutations at specific positions in the DNA sequence can trigger the onset of cancers and neurodegenerative diseases (Cantor, 2006; Collins, 2005; Cooke et al., 2003; Kamiya & Kasai, 1997; Kasai, 1997; Loft & Poulsen, 1996; Suzuki & Kamiya, 2016; Wu et al., 2004). This has created a need for methods to detect the presence and specific location of oxidatively damaged bases within DNA. Examples of purine nucleobases affected by oxidative damage include 8‐oxo‐2'‐deoxyguanosine (8‐oxo‐dG), 8‐oxo‐2'‐deoxyadenosine (8‐oxo‐dA), and 2‐hydroxy‐2'‐deoxyadenosine (2‐OH‐dA; also known as iso‐dG). For instance, 8‐oxo‐dG exhibits genotoxic properties, prompting the development of detection methods to investigate its occurrence and its links to disease. Recently, we successfully developed an artificial nucleic acid capable of specifically recognizing and detecting 8‐oxo‐dG in DNA (Aoki et al., 2020; Kikukawa et al., 2022; Taniguchi et al., 2011, 2015). Despite its very low concentration in DNA (fewer than 1 per 10 million normal nucleotides), 2‐OH‐dA shows a mutation rate of ∼0.8%, similar to 8‐oxo‐dG, and has been found in increased amounts in cancerous tissues (Jaruga et al., 1994; Kasai, 2002; Olinski et al., 1992; Satou et al., 2006). However, unlike 8‐oxo‐dG, 2‐OH‐dA cannot be detected using electrochemical sensors, making it difficult to confirm its presence in both its monomeric (2‐OH‐dATP) and DNA‐incorporated states. In response to this challenge, we developed an unnatural nucleoside, pseudo‐deoxycytidine (ψdC), which base pairs specifically with 2‐OH‐dA (iso‐dG) and can accommodate the tautomeric forms of the complementary base. Basic Protocol 1 details the synthesis of the phosphoramidite unit of the ψdC derivative and outlines its incorporation into oligodeoxynucleotides (ODNs) using an automated DNA synthesizer following standard protocols. It also covers the step‐by‐step synthesis of ψdC triphosphate (ψdCTP) (Figs. 1 and 2). Basic Protocol 2 describes the evaluation method for duplex formation between the synthesized ODN containing ψdC and an ODN containing 2‐OH‐dA (Fig. 3). Finally, Basic Protocol 3 addresses the process of incorporating ψdCTP into a primer strand using DNA polymerase with a template DNA containing 2‐OH‐dA (Fig. 4).

*CAUTION*: All reactions must be performed in a suitable fume hood with efficient ventilation with appropriate personal protective equipment (goggles, lab coat, and gloves).

*NOTE*: Throughout the text, the bolded numbers **1** to **16** and the text **X**, **Y**, **ODN1**, and **ODN2** indicate the compounds presented in Figure 1.

## Basic Protocol 1 SYNTHESIS OF ODNS HAVING ψdC AND ψdCTP

The synthesis of ψdC derivatives is shown in Figure 1. In the synthesis of ψdC, the known glycal (Cameron et al., 1997) was coupled with 5‐iodouracil under Mizorogi‐Heck reaction conditions, followed by desilylation and diastereoselective reduction to obtain **4**. The hydroxyl group was protected with a tert‐butyldimethylsilyl (TBS) group to yield **5**, and the endocyclic amino group was protected with a benzyl group to produce **6**. The carbonyl group at the 6‐position was activated with a 2,4,6‐triisopropylbenzenesulfonyl group and then treated with aqueous ammonia to convert it to an amino group, resulting in **8**. The benzyl group was removed to obtain **9**, and the endocyclic amino group was protected with a benzoyl group to yield **10**. To incorporate ψdC into ODNs, the TBS group was deprotected to obtain the diol **11**. The 5'‐hydroxyl group was protected with a 4,4'‐dimethoxytrityl (DMTr) group to yield **12**, and the 3'‐hydroxyl group was introduced into the phosphoramidite unit to produce **13**. The oligonucleotides (**ODN1** and **ODN2**) were synthesized using an automated DNA/RNA synthesizer according to conventional amidite chemistry. Nucleoside phosphoramidite reagents were used in this work. The ODNs were prepared with a DMTr‐on form, which were cleaved from the controlled pore glass (CPG) and deprotected by treatment with 28% aqueous ammonia for 24 hr at 55°C. The resulting crude mixture was treated with appropriate reagents to modify the aminoethyl group of the artificial nucleoside and purified by high‐performance liquid chromatography (HPLC) equipped with an octa decyl silyl (ODS) column using a linear gradient between 0.1 M triethylammonium acetate (TEAA) buffer and acetonitrile (CH₃CN). In Figure 2, the structures of the synthesized ODNs were identified by matrix‐assisted laser desorption/ionization time‐of‐flight mass spectrometry (MALDI‐TOF‐MS; negative mode) measurement. On the other hand, to synthesize the triphosphate, the 5'‐hydroxyl group of **10** was selectively deprotected to yield **14**. Nucleoside **14** was directly converted into the corresponding triphosphate **16** using Van Boom reagents under acidic conditions. After purification by HPLC, the new triphosphate was characterized by phosphorus nuclear magnetic resonance (NMR) and high‐resolution electrospray ionization‐mass spectrometry (HRESI‐MS).

### Materials

Palladium(II) acetate [Pd(OAc)_2_] (TCI Chemicals, cat. no. A1424)
Triphenylarsine (TCI Chemicals, cat. no. T0508)
Dry *N,N*‐dimethylformamide (DMF) (FUJIFILM Wako Pure Chemical, cat. no. 043‐32361)
Argon gas
Glycal **1** (Cameron et al., 1997)
5‐Iodo‐uracil (TCI Chemicals, cat. no. D4200)
Tributylamine (TBA) (TCI Chemicals, cat. no. T0357)
Hexane (FUJIFILM Wako Pure Chemical, cat. no. 083‐00417)
Ethyl acetate (EtOAc) (FUJIFILM Wako Pure Chemical, cat. no. 059‐00357)
Acetic acid (Nacalai tesque, cat. no. 00211‐95)
1.0 M tetrabutylammonium fluoride (TBAF) (TCI Chemicals, cat. no. T1125) in tetrahydrofuran (THF) (TCI, cat. no. T2394)
Dry dichloromethane (CH_2_Cl_2_) (FUJIFILM Wako Pure Chemical, cat. no. 042‐31231)
Methanol (MeOH) (FUJIFILM Wako Pure Chemical, cat. no. 139‐01827)
Chloroform (CHCl_3_) (FUJIFILM Wako Pure Chemical, cat. no. 036‐02607)
Dry acetonitrile (CH_3_CN) (FUJIFILM Wako Pure Chemical, cat. no. 018‐22901)
Sodium triacetoxyborohydride (TCI Chemicals, cat. no. S0394)
*tert*‐Butyldimethylsilyl chloride (TBSCl) (Nacalai tesque, cat. no. B0995)
Imidazole (TCI Chemicals, cat. no. I0001)
H_2_O, MilliQ
Saturated NaCl solution (brine) (see recipe)
Sodium sulfate (Na_2_SO_4_) (Nacalai tesque, cat. no. 31916‐15)
Sodium carbonate (TCI Chemicals, cat. no. S0560)
Benzyl bromide (TCI Chemicals, cat. no. B0411)
Saturated NaHCO_3_ solution (see recipe)
2,4,6‐Triisopropylbenzenesulfonyl chloride (TCI Chemicals, cat. no. T0459)
Triethylamine (TEA) (Nacalai tesque, cat. no. 34805‐62)
4‐Dimethylaminopyridine (DMAP) (TCI Chemicals, cat. no. D1450)
28% ammonia solution (Nacalai tesque, cat. no. 02512‐95)
Ammonium formate (Nacalai tesque, cat. no. 02509‐55)
10% palladium on carbon (Sigma‐Aldrich, cat. no. 205699)
Benzoyl chloride (TCI Chemicals, cat. no. B6723)
Pyridine (FUJIFILM Wako Pure Chemical, cat. no. 162‐05313)
Triethylamine trihydrofluoride (3HF‐TEA) (Sigma‐Aldrich, cat. no. 344648)
4, 4’‐Dimethoxytrityl chloride (DMTrCl) (TCI Chemicals, cat. no. D1612)
*N,N*‐Diisopropylethylamine (DIPEA) (TCI Chemicals, cat. no. D1599)
2‐Cyanoethyl *N*,*N*‐diisopropylchlorophosphoramidite (Sigma‐Aldrich, cat. no. M072000)
Bz‐dA‐CE phosphoramidite (Glen Research, cat. no. 10‐1000‐02)
iBu‐dG‐CE phosphoramidite (Glen Research, cat. no. 10‐1020‐02)
2.0 M triethylammonium acetate (TEAA) buffer (TCI Chemicals, cat. no. T4022)
∼70% hydrogen fluoride (HF) and ∼30% pyridine (TCI Chemicals, cat. no. P0999)
2‐chloro‐4H‐1,3,2‐benzodioxaphosphorin‐4‐one (TCI Chemicals, cat. no. C1210)
Dioxane (FUJIFILM Wako Pure Chemical, cat. no. 048‐03763)
Tributylammonium pyrophosphate (Sigma‐Aldrich, cat. no. P8533)
I_2_ (TCI Chemicals, cat. no. I0604)
5% (w/v) sodium hydrogen sulfite (NaHSO_3_) solution (see recipe)
Ethanol (EtOH) (FUJIFILM Wako Pure Chemical, cat. no. 057‐00456)
Sodium chloride (NaCl) (TCI Chemicals, cat. no. S0572)
Glycine (TCI Chemicals, cat. no. G0317)
Hydrochloric acid (HCl) (TCI Chemicals, cat. no. H1202)

20‐, 30‐, 50‐, 100‐, 200‐, 300‐, and 500‐ml round‐bottom flasks
Silica gel TLC plate Kiselgel 60 F_254_, 0.2‐mm (Merck, cat. no. 1.05715.0001)
UV lamp
Rotary evaporator
Flash column, SiOH, 50‐Å pore size, 10 g or 30 g (SHOKO Science, cat. nos. CAP04132 or CAP04133)
Smart Flash automated flash chromatograph system (Yamazen Corporation, cat. no. AI‐580S)
Vacuum oil pump
Oil bath
Filter paper (ADVANTEC, cat. no. 00011055)
Kiriyama Rohto funnel (KIRIYAMA Corporation, cat. no. S‐55)
Celite pad (Nacalai Tesque, cat. no. 08034‐85)
Reverse‐phase HPLC purification system with Nacalai Tesque COSMOSIL 5C18‐ARII 10 × 250–mm and 4.6 × 250–mm columns
Na^+^ form resin (Merck, cat. no. 44514)

Additional reagents and equipment for NMR and MS characterization (James, 2001)

### Coupling of glycal compound and 5‐iodouracil under the Mizorogi‐Heck reaction followed by desilylation and diastereoselective reduction

1 Add 418 mg palladium(II) acetate (1.9 mmol) and 1.1 g triphenylarsine (3.6 mmol) in 42 ml *N,N*‐dimethylformamide in a 200‐ml round‐bottom flask under an argon atmosphere.
2 Stir the mixture at room temperature for 30 min.
3 Add 3.3 g glycal **1** (9.6 mmol) (Cameron et al., 1997), 2.6 g of 5‐iodo‐uracil (11.0 mmol) and 1.8 ml tributylamine (19.2 mmol) in 42 ml *N,N*‐dimethylformamide.
4 Stir the mixture at 60°C for 24 hr.
5 Check the reaction by TLC.
  The product is visualized using a 254‐nm UV lamp. R_f_ = 0.89 for the product **2** and 0.02 for **1**, hexane/EtOAc (1:1, v/v).
6 Cool to room temperature.
7 Add 2.2 ml acetic acid and 19.2 ml tetrabutylammonium fluoride (1.0 M in THF, 19.2 mmol).
8 Stir the mixture at room temperature for 1 hr.
9 Check the reaction by TLC.
  The product is visualized using a 254‐nm UV lamp. R_f_ = 0.32 for the product **3** and 0.01 for **2**, CH_2_Cl_2_/MeOH (10:1, v/v).
10 Concentrate under vacuum using a rotary evaporator.
11 Purify by column chromatography (SHOKO 10 g, CH_2_Cl_2_/MeOH = 100:0 to 80:20) using a Smart Flash automated flash chromatograph system.
12 Dissolve the compound **3** with acetic acid/acetonitrile (7 ml/70 ml).
13 Add 3.0 g sodium triacetoxyborohydride (14.4 mmol) to this solution in a 500‐ml round‐bottom flask at 0°C under an argon atmosphere.
14 Stir the mixture at room temperature for 2 hr.
15 Check the reaction by TLC.
  The product is visualized using a 254‐nm UV lamp. R_f_ = 0.01 for the product **4** and 0.18 for **3**, CH_2_Cl_2_/MeOH (10:1, v/v).
16 Concentrate under vacuum using a rotary evaporator.
17 Purify by column chromatography (SHOKO 30 g, CHCl_3_/MeOH = 100:0 to 80:20) using a Smart Flash automated flash chromatograph system.
18 Characterize the compound by ^1^H‐NMR, ^13^C‐NMR, and HRESI‐MS.
  The pure compound **4** is obtained as a white powder (1.1 g, 4.8 mmol, 53% for 3 steps). ^1^H‐NMR (500 MHz, DMSO) δ 11.03 (1H, s), 7.40 (1H, s), 5.00‐4.97 (1H, m), 4.35‐4.30 (1H, m), 4.15‐4.08 (1H, m), 3.71‐3.66 (1H, m), 3.41‐3.39 (1H, m), 2.03‐1.09 (1H, m), 1.79‐1.71 (1H, m); ^13^C NMR (125 MHz, DMSO) δ 163.5, 151.1, 137.9, 113.0, 87.1, 73.2, 72.1, 62.2, 45.5; HRMS (ESI‐TOF) calculated for C_9_H_12_N_2_O_5_Na [M+Na]^+^: 251.0638, found: 251.0649.

### Protecting the 3',5'‐hydroxyl groups in 4

19 Add 2.1 g *tert*‐butyldimethylsilyl chloride (14.3 mmol), 1.3 g imidazole (19.1 mmol) and 1.1 g of **4** (4.78 mmol) to the solution of 9.6 ml *N,N*‐dimethylformamide in a 100‐ml round‐bottom flask under an argon atmosphere.
20 Stir the mixture at room temperature for 3 hr.
21 Check the reaction by TLC.
  The product is visualized using a 254‐nm UV lamp. R_f_ = 0.88 for the product **5** and 0.01 for **4**, CH_2_Cl_2_/MeOH (10:1, v/v).
22 Add 32 ml hexane and 8 ml EtOAc and wash with 30 ml H_2_O and 30 ml brine.
23 Dry the separated organic layer using Na_2_SO_4_, filtere using filter paper with a Kiriyama Rohto funnel, and concentrate under vacuum using a rotary evaporator.
24 Purify by column chromatography (SHOKO 10 g, hexane/EtOAc = 80/20 to 60/40) using a Smart Flash automated flash chromatograph system.
25 Characterize the compound by ^1^H‐NMR, ^13^C‐NMR, and HRESI‐MS.
  The pure compound **5** is obtained as a white powder (1.6 g, 3.4 mmol, 72%). (^1^H‐NMR (500 MHz, CDCl_3_) δ 10.16 (1H, s), 9.70 (1H, s), 7.48 (1H, s), 5.06‐4.98 (1H, m), 4.35‐4.30 (1H, m), 3.90‐3.84 (1H, m), 3.68 (1H, dd, *J* = 3.5, 11 Hz), 3.58 (1H, dd, *J* = 5.5, 11 Hz), 2.32‐2.25 (1H, m), 1.86‐1.77 (1H, m), 0.88 (18H, s), 0.10‐0.04 (12H, m); ^13^C NMR (125 MHz, CDCl_3_) δ 162.9, 152.5, 136.7, 115.9, 87.0, 76.8, 73.2, 63.2, 41.0, 25.5, 18.0, ‐5.00; HRMS (ESI‐TOF) calculated for C_21_H_40_N_2_O_5_Si_2_Na [M+Na]^+^: 479.2367, found: 479.2367.

### Protecting the endocyclic amino group in 5

26 Add 0.7 g sodium carbonate (6.9 mmol) and 1.6 g of **5** (3.4 mmol) to the solution of 69 ml *N,N*‐dimethylformamide in a 200‐ml round‐bottom flask under an argon atmosphere.
27 Stir the mixture at room temperature for 1 hr.
28 Cool to 0°C.
29 Add 0.48 ml benzyl bromide (3.44 mmol) to the reaction mixture.
30 Stir the mixture at room temperature for 24 hr.
31 Check the reaction by TLC.
  The product is visualized using a 254‐nm UV lamp. R_f_ = 0.21 for the product **6** and 0.05 for **5**, hexane/EtOAc (3:1, v/v).
32 Add 160 ml hexane and 40 ml EtOAc and wash with 200 ml saturated NaHCO_3_ aqueous solution, 200 ml H_2_O and 200 ml brine.
33 Dry the separated organic layer using Na_2_SO_4_, filter using filter paper with a Kiriyama Rohto funnel, and concentrate under vacuum using a rotary evaporator.
34 Purify by column chromatography (SHOKO 10 g, Hexane/EtOAc = 80:20 to 10:90) using a Smart Flash automated flash chromatograph system.
35 Characterize the compound by ^1^H‐NMR, ^13^C‐NMR, and HRESI‐MS.
  The pure compound **6** is obtained as a white powder (1.2 g, 2.27 mmol, 66%). (^1^H‐NMR (500 MHz, CDCl_3_) δ 9.79 (s, 1H), 7.37‐7.26 (m, 6H), 5.01‐4.96 (1H, m), 4.88‐4.85 (2H, m), 4.31‐4.26 (1H, m), 3.86‐3.80 (1H, m), 3.62‐3.54 (1H, m), 3.53‐3.47 (1H, m), 2.31‐2.25 (1H, m), 1.76‐1.68 (1H, m), 0.96‐0.75 (18H, m), 0.12‐0.02 (12H, m); ^13^C NMR (125 MHz, CDCl_3_) δ 162.7, 151.4, 139.7, 135.6, 129.1, 116.5, 87.5, 77.4, 73.8, 63.6, 51.5, 41.6, 25.9, 18.3, ‐4.7; HRMS (ESI‐TOF) calculated for C_28_H_47_N_2_O_5_Si_2_ [M+H]^+^: 547.3018, found: 547.3035.

### Conversion of the 6‐position carbonyl group to an amino group

36 Add 1.2 g of **6** (2.3 mmol) to the solution of 100 ml acetonitrile at 0°C in a 300‐ml round‐bottom flask under an argon atmosphere.
37 Add 1.4 g of 2,4,6‐triisopropylbenzenesulfonyl chloride (4.5 mmol), 1.2 ml triethylamine (9.1 mmol), and 0.28 g of 4‐dimethylaminopyridine (2.3 mmol) to the reaction mixture at 0°C.
38 Stir the mixture at room temperature for 24 hr.
39 Check the reaction by TLC.
  The product is visualized using a 254‐nm UV lamp. R_f_ = 0.63 for the product **7** and 0.24 for **6**, hexane/EtOAc (1:1, v/v).
40 Add 7 ml of 28% ammonia solution to the reaction mixture.
41 Stir the mixture at room temperature for 4 hr.
42 Check the reaction by TLC.
  The product is visualized using a 254‐nm UV lamp. R_f_ = 0.54 for the product **8** and 0.79 for **7**, CH_2_Cl_2_/MeOH (10:1, v/v).
43 Add 200 ml dichloromethane and wash with 100 ml brine.
44 Dry the organic layer over Na_2_SO_4_, filter with a Kiriyama Rohto funnel, and concentrate under vacuum using a rotary evaporator.
45 Purify by column chromatography (SHOKO 10 g, hexane/EtOAc = 80:20 to 10:90) using a Smart Flash automated flash chromatograph system.
46 Characterize the compound by ^1^H‐NMR, ^13^C‐NMR, and HRESI‐MS.
  The pure compound **8** is obtained as a white foam (0.88 g, 1.6 mmol, 71%). ^1^H‐NMR (500 MHz, CDCl_3_) δ 7.43 (1H, s), 7.34‐7.26 (5H, m), 5.12 (1H, d, *J* = 14.5 Hz), 4.92 (1H, d, *J* = 14.5 Hz), 4.84 (1H, dd, *J* = 5.0, 11 Hz), 4.39‐4.34 (1H, m), 3.89‐3.87 (1H, m), 3.85‐3.80 (1H, m), 3.76‐3.71 (1H, m), 2.24‐2.21 (1H, m), 1.89‐1.83 (1H, m), 0.92‐0.84 (18H, m), 0.10‐0.02 (12H, m); ^13^C NMR (125 MHz, CDCl_3_) δ 163.8, 156.2, 143.3, 136.3, 128.9, 128.0, 104.3, 88.5, 77.6, 73.8, 63.3, 52.1, 40.9, 22.9, 18.5, ‐4.7; HRMS (ESI‐TOF) calculated for C_28_H_48_N_3_O_4_Si_2_ [M+H]^+^: 546.3178, found: 546.3219.

### Remove the 1‐benzyl group in 8

47 Add 0.20 g ammonium formate (3.2 mmol) and 66.7 mg of 10% palladium‐activated carbon to a solution of 0.88 g of **8** (1.6 mmol) in 21 ml methanol in a 100‐ml round‐bottom flask under an argon atmosphere.
48 Stir the mixture at room temperature for 24 hr.
49 Check the reaction by TLC.
  The product is visualized using a 254‐nm UV lamp. R_f_ = 0.40 for the product **9** and 0.91 for **8**, CH_2_Cl_2_/MeOH (10:1, v/v).
50 Filter the mixture with Celite pad and concentrate under vacuum using a rotary evaporator.
51 Purify by column chromatography (SHOKO 10 g, CDCl_3_/MeOH = 100:0 to 90:10) using a Smart Flash automated flash chromatograph system.
52 Characterize the compound by ^1^H‐NMR, ^13^C‐NMR, and HRESI‐MS.
  The pure compound **9** is obtained as a yellow oil (0.64 g, 1.4 mmol, 88%). (^1^H‐NMR (500 MHz, CDCl_3_) δ 7.69 (1H, s), 5.14‐5.00 (1H, m), 4.92 (1H, d, *J* = 14.5 Hz), 4.65‐4.62 (1H, m), 4.11‐4.08 (1H, m), 3.89‐3.87 (1H, m), 4.09 (1H, dd, *J* = 2.5, 5 Hz), 4.00‐3.98 (2H, m), 2.40‐2.31 (1H, m), 2.17‐2.13 (1H, m), 1.15‐1.10 (18H, m), 0.30‐0.20 (12H, m); ^13^C NMR (125 MHz, CDCl_3_) δ 163.4, 152.7, 137.2, 116.0, 87.2, 76.7, 73.4, 63.4, 41.8, 25.8, 18.3, ‐5.3; HRMS (ESI‐TOF) calculated for C_21_H_42_N_3_O_4_Si_2_ [M+H]^+^: 456.2708, found: 456.2753.

### Benzoylation of the amino group in 9

53 Add 0.39 g benzoyl chloride (2.8 mmol) to the solution of 0.64 g of **9** (1.4 mmol) in 26 ml pyridine at 0°C in a 100‐ml round‐bottom flask under an argon atmosphere.
54 Stir the mixture at room temperature for 2 hr.
55 Check the reaction by TLC.
  The product is visualized using a 254‐nm UV lamp. R_f_ = 0.78 for the product **10** and 0.28 for **9**, CH_2_Cl_2_/MeOH (10:1, v/v).
56 Add 100 ml dichloromethane and wash with 100 ml brine.
57 Dry the separated organic layer using Na_2_SO_4_, filter using filter paper with a Kiriyama Rohto funnel, and concentrate under vacuum using a rotary evaporator.
58 Purify by column chromatography (SHOKO 30 g, Hexane/EtOAc = 80:20 to 10:90) using a Smart Flash automated flash chromatograph system. Characterize the compound by ^1^H‐NMR, ^13^C‐NMR, and HRESI‐MS.
  The pure compound **10** is obtained as a yellow foam (0.6 g, 1.1 mmol, 78%). (^1^H‐NMR (500 MHz, CDCl_3_) δ 13.43 (1H, s), 10.73 (1H, s), 8.30‐8.25 (2H, m), 7.64 (1H, s), 7.55‐7.50 (1H, m), 7.45‐7.39 (2H, m), 5.34 (1H, dd, *J* = 5.5, 9.0 Hz), 4.40‐4.36 (1H, m), 4.65‐4.62 (1H, m), 3.99‐3.94 (1H, m), 3.73 (1H, dd, *J* = 4.0, 11.0 Hz), 3.59 (1H, dd, *J* = 5.5, 11.0 Hz), 2.61‐2.53 (1H, m), 1.76‐1.66 (1H, m), 1.00‐0.89 (18H, m), 0.14‐0.07 (12H, m); ^13^C NMR (125 MHz, CDCl_3_) δ 179.5, 158.7, 150.3, 137.3, 132.6, 129.9, 128.1, 117.2, 87.4, 76.7, 73.8, 63.8, 42.5, 25.9, 18.3, ‐5.3; HRMS (ESI‐TOF) calculated for C_28_H_46_N_3_O_5_Si_2_ [M+H]^+^: 560.2971, found: 560.3019.

### Remove the 3’,5’‐tert‐butylsilyl group in 10

59 Add 684 mg triethylamine trihydrofluoride (4.3 mmol) and 600 mg of **10** (1.1 mmol) to the solution of 28 ml tetrahydrofuran in a 100‐ml round‐bottom flask under an argon atmosphere.
60 Stir the mixture at room temperature overnight.
61 Check the reaction by TLC.
  The product is visualized using a 254‐nm UV lamp. R_f_ = 0.12 for the product **11** and 0.84 for **10**, CH_2_Cl_2_/MeOH (10:1, v/v)).
62 Concentrate under vacuum using a rotary evaporator.
63 Purify by column chromatography (SHOKO 10 g, CDCl_3_/MeOH = 100:0 to 90:10) using a Smart Flash automated flash chromatograph system.
64 Characterize the compound by ^1^H‐NMR, ^13^C‐NMR, and HRESI‐MS.
  The pure compound **11** is obtained as a white powder (278 mg, 0.8 mmol, 81%). ^1^H‐NMR (500 MHz, DMSO) δ 13.02 (1H, s), 11.07 (1H, s), 8.27‐8.12 (2H, m), 7.78 (1H, s), 7.63‐7.55 (1H, m), 7.53‐7.44 (2H, m), 5.20‐5.12 (1H, m), 4.84‐4.76(1H, m), 4.17‐4.11 (1H, m), 3.87‐3.72 (1H, m), 3.50‐3.40 (2H, m), 2.42‐2.32 (1H, m), 1.81‐1.72 (1H, m); ^13^C NMR (125 MHz, DMSO) δ 179.5, 158.9, 150.0, 138.6, 132.6, 129.9, 128.1, 116.3, 87.7, 76.8, 73.7, 62.9, 43.2, 25.7, 17.9, ‐4.7; HRMS (ESI‐TOF) calculated for C_16_H_18_N_3_O_5_ [M+H]^+^: 332.1241, found: 332.1282.

### Dimethoxytritylation of the 5’‐hydroxyl group in 11

65 Add 540 mg of 4,4‐dimethoxytrityl chloride (1.7 mmol) and 278 mg of **11** (0.8 mmol) to the solution of 28 ml pyridine in a 100‐ml round‐bottom flask under an argon atmosphere.
66 Stir the mixture at room temperature for 2 hr.
67 Check the reaction by TLC.
  The product is visualized using a 254‐nm UV lamp. R_f_ = 0.51 for the product **12** and 0.20 for **11**, CH_2_Cl_2_/MeOH (10:1, v/v).
68 Add 16 ml hexane and 50 ml EtOAc and wash with 20 ml saturated NaHCO_3_ aqueous solution and 20 ml brine.
69 Dry the separated organic layer using Na_2_SO_4_, filtered using filter paper with a Kiriyama Rohto funnel, and concentrate under vacuum using a rotary evaporator.
70 Purify by column chromatography (SHOKO 30 g, CH_2_Cl_2_/MeOH = 100:0 to 90:10) using a Smart Flash automated flash chromatograph system.
71 Characterize the compound by ^1^H‐NMR, ^13^C‐NMR, and HRESI‐MS.
  The pure compound **12** is obtained as a yellow foam (143 mg, 0.2 mmol, 27%). ^1^H‐NMR (500 MHz, CDCl_3_) δ 13.32 (1H, s), 9.54 (1H, s), 8.36‐8.18 (2H, m), 7.63‐7.55 (2H, m), 7.48‐7.32 (4H, m), 7.35‐7.20 (9H, m), 6.88‐6.80 (4H, m), 5.35‐5.30 (1H, t, *J* = 7.0 Hz), 4.43‐4.39 (1H, m), 4.08‐4.04 (1H, dd, *J* = 4.0, 8.5 Hz), 3.81‐3.73 (6H, m), 3.36 (1H, dd, *J* = 4.5, 10 Hz), 3.30 (1H, dd, *J* = 4.5, 10 Hz), 2.67‐2.60 (1H, m), 1.75‐1.50 (1H, m); ^13^C NMR (125 MHz, CDCl_3_) δ 179.3, 172.3, 158.4, 144.7, 136.1, 129.8, 129.1, 128.5, 128.3, 127.9, 127.8, 123.7, 113.5, 86.6, 85.43, 77.0, 73.3, 64.3, 55.1, 42.2; HRMS (ESI‐TOF) calculated for C_37_H_36_N_3_O_7_ [M+H]^+^: 656.2367, found: 656.2393.
  This dimethoxytritylation yield is low, but ∼40% of compound **11** remains to be recovered.

### Phosphoramidation of the 3'‐hydroxyl group in 12

72 Add 0.2 g *N,N*‐diisopropylethylamine (1.4 mmol) and 130 mg of 2‐cyanoethyl‐*N*,*N*‐diisopropylchlorophosphoramidite (0.6 mmol) to the solution of the 143 mg of **12** (0.2 mmol) in 4.6 ml dichloromethane at 0°C in a 20‐ml round‐bottom flask under an argon atmosphere.
73 Stir the mixture at room temperature for 4 hr.
74 Check the reaction by TLC.
  The product is visualized using a 254‐nm UV lamp. R_f_ = 0.33 for the product **13** and 0.10 for **12**, hexane/EtOAc (3:1, v/v)).
75 Add 16 ml hexane and 10 ml EtOAc and wash with 5 ml brine.
76 Dry the separated organic layer using Na_2_SO_4_ and concentrate using a rotary evaporator.
77 Purify by column chromatography (SHOKO 10 g, hexane/EtOAc = 60:40) using a Smart Flash automated flash chromatograph system.
78 Characterize the compound by ^1^H‐NMR, ^13^C‐NMR, and HRESI‐MS.
79 The foam is reprecipitated with hexane at –78°C.
  The pure compound **13** is obtained as the yellow oil (70 mg, 0.1 mmol, 37%). ^1^H‐NMR (500 MHz, CDCl_3_) δ 7.48‐7.39 (2H, m), 7.38‐7.27 (12H, m), 6.88‐6.80 (6H, m), 5.35‐5.30 (1H, t, *J* = 7.0 Hz), 4.69‐4.47 (1H, m), 4.26‐4.03 (1H, m), 3.88‐3.73 (9H, m), 3.64‐3.44 (2H, m), 3.21‐3.12 (1H, m), 3.05‐2.99 (1H, m), 2.68‐2.52 (1H, m), 2.12‐2.03 (1H, m), 1.26‐1.02 (12H, m); ^31^P NMR (202 MHz, CDCl_3_) δ 147.9; HRMS (ESI‐TOF) calculated for C_46_H_52_N_5_O_8_PNa [M+Na]^+^: 856.3446, found: 856.3458.
  This phosphoramidation yield is low, the cause is unknown and unidentifiable compounds are formed.

### Oligonucleotides synthesis containing ψdC derivatives

#### Incorporation of amidite *unit 13* into ODNs

80 Synthesize 13‐mer oligonucleotides (**ODN1**: 5’‐CTTTCT **X** CTCCTT‐3’, **ODN2**: 5’‐AAGGAG **Y** AGAAAG‐3’, **X** = **Y** = ψdC) on an automated DNA synthesizer (Nihon Techno Service Co.) using Bz‐dA‐CE phosphoramidite, iBu‐dG‐CE phosphoramidite and standard phosphoramidite chemistry in 1‐µmol scale.
81 Cleavage from the resin was accomplished by an overnight treatment with 28% ammonium hydroxide at 55°C, then remove the solvent in vacuo.
82 Purify by reverse‐phase HPLC purification using a Nacalai Tesque COSMOSIL 5C18‐ARII 10 × 250–mm column as follows:
  - Solvent A, 0.1 M TEAA buffer.
  - Solvent B, CH_3_CN.
  - Gradient, solvent B from 10% to 40% for 20 min.
  - Flow rate, 3.0 ml/min.
  - UV detector, 254 nm.
  - Column oven, 35°C.
83 The DMTr group was removed in 5% acetic acid aqueous solution at room temperature for 30 min.
84 Purify by reverse‐phase HPLC purification using a Nacalai Tesque COSMOSIL 5C18‐ARII 4.6 × 250–mm column as follows:
  - Solvent A, 0.1 M TEAA buffer.
  - Solvent B, CH_3_CN.
  - Gradient, solvent B from 5% to 30% for 20 min.
  - Flow rate, 1.0 ml/min.
  - UV detector, 254 nm.
  - Column oven, 35°C.
85 The structural integrity of synthesized ODNs, including ψdC, was analyzed by MALDI‐TOF MS. The isolated yield of **ODN1** and **ODN2** having ψdC was 13.7 and 7.7 ODU, respectively.

### Remove the 5’‐tert‐butylsilyl group in 10

86 Add 155 mg of **10** (0.28 mmol) to 3.0 ml tetrahydrofuran and cooled to 0°C in a 20‐ml round‐bottom flask under an argon atmosphere.
87 Add 31 mg of hydrogen fluoride pyridine (∼70% HF and ∼30% pyridine) (0.85 mmol) to the reaction mixture at 0°C.
88 Stir the mixture at room temperature for 24 hr.
89 Check the reaction by TLC.
  The product is visualized using a 254‐nm UV lamp. R_f_ = 0.26 for the product **14** and 0.92 for **10**, CH_2_Cl_2_/MeOH (10:1, v/v).
90 Add 16 ml hexane and 10 ml EtOAc and wash with 5 ml saturated NaHCO_3_ aqueous solution.
91 Dry the separated organic layer using Na_2_SO_4_ and concentrate under vacuum using a rotary evaporator.
92 Purify by column chromatography (SHOKO 10 g, CDCl_3_/MeOH = 100:0 to 80:20) using a Smart Flash automated flash chromatograph system.
93 Characterize the compound by ^1^H‐NMR, ^13^C‐NMR, and HRESI‐MS.
  The pure compound **14** is obtained as a yellow foam (21.2 mg, 0.05 mmol, 18%). ^1^H‐NMR (500 MHz, CDCl_3_) δ 13.36 (1H, s), 11.07 (1H, s), 8.30‐8.12 (2H, m), 7.88 (1H, s),7.53‐7.46 (1H, m), 7.44‐7.32 (2H, m), 5.37‐5.31 (1H, m), 4.38‐4.23 (1H, m), 4.65‐4.62 (1H, m), 4.00‐3.87 (1H, m), 3.88‐3.75 (1H, m), 3.74‐3.50 (1H, m), 2.60‐2.38 (1H, m), 1.87‐1.69 (1H, m), 1.01‐0.78 (9H, m), 0.13‐0.03(6H, m); ^13^C NMR (125 MHz, CDCl_3_) δ 179.5, 158.9, 150.0, 138.6, 132.6, 129.9, 128.1, 116.3, 87.7, 76.8, 73.7, 62.9, 43.2, 25.7, 17.9, ‐4.7; HRMS (ESI‐TOF) calculated for C_22_H_31_N_3_O_5_SiNa [M+Na]^+^: 468.1975, found: 468.1925.
  This selective deprotection yield is low, but ∼50% of compound **10** is recovered.

### Triphosphorylation of the 5'‐hydroxyl group and remove the 3’‐tert‐butylsilyl group in 14

94 Add 11.5 mg of 2‐chloro‐4*H*‐1,3,2‐benzodioxaphosphorin‐4‐one (57 µmol) in 200 µl dioxane to a solution of compound 12.4 mg of **14** (28 µmol) in 200 µl pyridine/dioxane (1:1) in a 10‐ml round‐bottom flask under an argon atmosphere.
95 Stir the mixture at room temperature for 30 min.
96 Check the reaction by TLC.
  The product is visualized using a 254‐nm UV lamp. R_f_ = 0.01 for the product **15** and 0.46 for **14**, CH_2_Cl_2_/MeOH (10:1, v/v).
97 Add 45 mg tributylammonium pyrophosphate (84 µmol) in 150 µl DMF and 50 µl tributylamine (0.21 mmol) to the reaction mixture.
98 Stir the mixture at room temperature for 30 min.
99 Add 1.0 ml of 1% I_2_ solution to the reaction mixture.
100 Stir the mixture at room temperature for 30 min.
101 Add 0.65 ml of 5% aqueous NaHSO_3_ solution to the reaction mixture.
102 Stir the mixture at room temperature for 1 hr.
103 Concentrate under vacuum using a rotary evaporator.
104 Lyophilize under a vacuum.
105 Wash the residue with 2 ml of 75% ethanol in water including 0.07 M NaCl, and the dissolve the precipitate in 4 ml water.
106 Purify by reverse‐phase HPLC purification using a Nacalai Tesque COSMOSIL 5C18‐ARII 10 × 250–mm column as follows:
  - Solvent A, 20 mM TEAA buffer.
  - Solvent B, CH_3_CN.
  - Linear gradient, solvent B from 20% to 60% for 40 min.
  - Flow rate, 3.0 ml/min.
  - UV detector, 254 nm.
  - Column oven, 35°C.
107 After lyophilization, dissolve the residue in 28% ammonia solution.
108 Stir the mixture at room temperature overnight.
109 Add 500 µl of 0.1 M glycine‐HCl buffer, pH 2.5, to the mixture.
110 Stir the mixture at 30°C for 4 hr.
111 Add 500 µl water to solution of mixture and wash with 200 µl hexane.
112 Purify the aqueous layer by reverse‐phase HPLC purification using a Nacalai Tesque COSMOSIL 5C18‐ARII 10 × 250–mm column as follows:
  - Solvent A, 20 mM TEAA buffer.
  - Solvent B, CH_3_CN.
  - Linear gradient, solvent B from 0% to 15% for 20 min.
  - Flow rate, 3.0 ml/min.
  - UV detector, 254 nm.
  - Column oven, 35°C.
113 After treating with Na^+^ form resin, characterize the compound by ^1^H‐NMR, and HRESI‐MS.
  After lyophilization, the pure compound **16** is obtained as a white powder (1.96 µmol, 7%) was obtained. ^1^H‐NMR (500 MHz, D_2_O) δ 7.60 (1H, s), 5.06‐5.00 (1H, m), 4.64‐4.59 (1H, m), 4.19‐4.11 (3H, m), 2.40‐2.29 (1H, m), 2.15‐2.09 (1H, m); ^31^P NMR (202 MHz, CDCl_3_) δ ‐5.69, ‐10.63, ‐20.80; HRMS (ESI‐TOF) calculated for C_9_H_15_N_3_O_13_P_3_ [M‐H]^‐^: 465.9812, found: 465.9849.
  This triphosphorylation yield is low because HPLC purification is performed twice in this reaction.

## Basic Protocol 2 MELTING TEMPERATURE OF DUPLEX FORMATION BETWEEN ODNS CONTAINING ψdC UNIT AND 2‐OH‐dA

The melting temperatures (T_m_ values) were measured to assess the nucleobase selectivity and stability of unnatural base pairing, including the novel ψdC, within the 13‐mer duplex DNA formed by the **ODN1** and **ODN2** sequences. T_m_ values were determined in a solution comprising each ODN strand at a concentration of 3 µM, along with 100 mM NaCl, 7.5 mM MgCl_2_, and 5 mM sodium phosphate buffer at pH 6.9. The data were processed using a melt curve analysis program. T_m_ values were calculated as the average from three or more independent experiments, with a precision of ±0.5°C (Fig. 3).

### Materials

13‐mer oligonucleotides having ψdC (see Basic Protocol 1)
DNA having 2‐OH‐dA (see Basic Protocol 1)
2× melting temperature buffer (see recipe)

UV‐visible spectrophotometer (JASCO, cat. no. V‐730BIO)

### Melting temperatures of ODN chains containing 2‐OH‐dA and ODN chains containing ψdC

1 Mix the 3 µM 13‐mer oligonucleotides having ψdC (ODN1: 5’‐CTTTCT **X** CTCCTT‐3’, ODN2: 5’‐AAGGAG **Y** AGAAAG‐3’, **X** = **Y** = ψdC) with DNA having 2‐OH‐dA (ODN1: 5’‐CTTTCT **X** CTCCTT‐3’, ODN2: 5’‐AAGGAG **Y** AGAAAG‐3’, **X** = **Y** = 2‐OH‐dA) in melting temperature buffer containing 100 mM NaCl, 7.5 mM MgCl_2_, and 5 mM sodium phosphate buffer at pH 6.9.
2 Measure the absorbance at 260 nm using a spectrophotomter as the temperature iss increased by 1°C/min from 20° to 80°C.

## Basic Protocol 3 A SINGLE NUCLEOTIDE PRIMER EXTENSION REACTION OF ψdCTP FOR A TEMPLATE STRAND CONTAINING 2‐OH‐dA

A single nucleotide primer extension reaction was performed using Klenow fragment (exo^–^) to elucidate the base selectivity of ψdCTP in a template sequence containing 2‐OH‐dA, dA, dG, dC, or T. When ψdCTP was used, primer extension was observed when 2‐OH‐dA was present at the complementary position of the template strand. In contrast, no significant extension was detected with template strands containing other bases under these conditions. This indicates that ψdCTP is selectively incorporated into the primer strand opposite 2‐OH‐dA during the polymerase reaction (Fig. 4).

### Materials

Template (Z) (final concentration 1.0 µM, 25‐mer, 5’‐CGACAGTTA Z GGTTAGGGTTATGCG‐3’; Z = 2‐OH‐dA, dC, dG, T, or dA (see Basic Protocol 1)
Primer (final concentration 1.0 µM, FAM‐labeled 15‐mer primer, 5’‐FAM‐CGCATAACCCTAACC‐3’)
dNTPs (New England Biolabs, cat. no. N0447S)
Klenow fragment (exo^−^) (New England Biolabs, cat. no. M0212S)
NEBuffer 2 (New England Biolabs, cat. no. B7002S) consisting of:
  10 mM Tris‐HCl
  50 mM NaCl
  10 mM MgCl_2_
  1 mM DTT
  pH 7.9
Loading buffer (Thermo Fisher Scientific, cat. no. AM8546G)
20% (w/v) denaturing polyacrylamide gel (see recipe)

0.6‐ml microtube (Wastson Bio Lab, cat. no. 130‐806C)
Dry block bath (EYELA, cat. no. MG‐2200)
Lumino‐image analyzer (FUJIFILM, cat. no. LAS‐4000)

### Incorporation reaction into the template strand containing 2‐OH‐dA using ψdCTP

1 Anneal 1.0 µM template (Z) and 1.0 µM primer in NEBuffer 2 in a microtube at 90°C for 5 min using a dry block bath.
2 Add the corresponding dNTPs (final concentration 25 µM in a 10 µl reaction volume) and 1.0 U Klenow fragment (exo^–^).
3 Incubate the mixture 1 min at 37°C using a dry block bath.
4 Quench the reaction with loading buffer and analyze by 20% denaturing polyacrylamide gel electrophoresis at room temperature for 2 hr.
5 Visualize bands using an image analyzer.

## Reagents and Solutions

### Denaturing polyacrylamide gel, 20% (w/v)

37.5 ml of 40% (w/v) acrylamide/bis mixed solution (19:1) (Nacalai, cat. no. 06140‐45), 20% (w/v) final
7.5 ml Tris‐borate‐EDTA buffer (Nacalai, cat. no. 35440‐31)
31.5 g urea (Nacalai, cat. no. 35907‐44), 7 M final
7.5 ml ddH_2_O
Store up to 1 year at room temperature

### Melting temperature buffer, 2×

15 µl of 1 M magnesium chloride (MgCl) (Wako Pure Chemical, cat. no. 310‐90361), 15 mM final
100 µl of 100 mM phosphate buffer solution (Nacalai, cat. no. 08968‐81), 10 mM final
40 µl of 5 M sodium chloride (NaCl) solution (Nacalai, cat. no. 06900‐14), 200 mM final
845 µl ddH_2_O
Store up to 1 year at room temperature

### NaCl solution, saturated (brine)

>37 g sodium chloride (NaCl) (TCI Chemicals, cat. no. S0572)
100 ml ddH_2_O
Store up to 1 year at room temperature

### NaHSO_3_ solution, 5% (w/v)

5.0 g sodium hydrogen sulfite (NaHSO_3_) (FUJIFILM Wako Pure Chemical, cat. no. 197‐01385)
100 ml ddH_2_O
Store up to 1 year at room temperature

### NaHCO_3_ solution, saturated

>12 g sodium hydrogen carbonate (NaHCO_3_) (TCI, cat. no. S0561)
100 ml ddH_2_O
Store up to 1 year at room temperature

## Commentary

### Background Information

The canonical features of DNA are important for the maintenance of biological activity. However, they are constantly damaged by external factors, such as radiation, ultraviolet rays, and chemical substances, and internal factors, including reactive oxygen species generated during metabolism. Among the many damaged nucleic acids, oxidatively damaged bases may pair with non‐complementary bases, leading to transversion mutations (Cooke et al., 2003; Kamiya & Kasai, 1997; Suzuki & Kamiya, 2016). Although this damage is generally random, a mutation at a specific position in the DNA sequence may lead to the development of cancer or neurodegenerative diseases (Cantor, 2006; Collins, 2005; Loft & Poulsen, 1996; Kasai, 1997; Wu et al., 2004). Therefore, a method is needed to detect the location of oxidatively damaged bases in the DNA sequence. Typical examples of oxidatively damaged purine nucleobases are 8‐oxo‐2’deoxyguanosine (8‐oxo‐dG) and 2‐hydroxy‐2’‐deoxyadenosine (2‐OH‐dA; also called iso‐dG). However, in contrast to 8‐oxo‐dG, 2‐OH‐dA is undetectable with an electrochemical detector. Therefore, its presence in the monomeric state, 2‐OH‐dATP, and in DNA is challenging.

We focused on the detection of 2‐OH‐dA in DNA and developed an unnatural nucleoside that forms a specific base pair with 2‐OH‐dA (iso‐dG). Since 2‐OH‐dA has a tautomer, we designed 2’‐deoxy‐pseudocytidine (ψdC) to enable the recognition of its structure (Miyahara & Taniguchi, 2022). We herein focused on the use of ψdC derivatives to recognize and detect 2‐OH‐dA in DNA by duplex formation and primer extension reactions.

The results of the melting temperature measurements show that the melting curve is highest when **ODN1** has ψdC and **ODN2** has 2‐OH‐dA on the complementary strand, indicating that the most stable duplex DNA is formed (Fig. 3, left). Similarly, the highest melting curve for the sequence with ψdC in **ODN2** was also observed when 2‐OH‐dA was present in the complementary strand in **ODN1** (Fig. 3, right). Moreover, we tested the single nucleotide primer extension reaction using Klenow Fragment (exo^–^) to clarify the nucleobase selectivity of ψdCTP in the template sequence containing 2‐OH‐dA, dA, dG, dC, or T. When ψdCTP was used, the primer extension reaction was only observed with the template strand containing 2‐OH‐dA (Fig. 4). We, therefore, developed ψdC, an artificial nucleoside with excellent recognition ability for 2‐OH‐dA (Figs. 3 and 4), and its synthesis is described in this article. The protocol described here uses commonly available reagents and can be performed without the need for complicated equipment or manipulation.

### Critical Parameters and Troubleshooting

In the synthesis of compound **6**, it is important to carefully control the equivalent amount of Na₂CO₃ used (Table 1). If the amount of base is not appropriate, the yield of the target compound will be reduced due to a much higher remaining amount of starting material or the formation of dibenzyl compounds. In the synthesis of compound **16**, if the reaction time after the addition of tributylammonium pyrophosphate exceeds 30 min, the yield of the target compound may decrease due to an increase in by‐products.

**Table 1:** Troubleshooting Guide for Synthesis of ψdC Derivatives

Problem	Possible cause	Solution
Poor yield	Formation of dibenzyl compounds	Use 2 equivalents of sodium carbonate
	Degradation of intermediates	Perform reaction in <30 min

### Understanding Results

This article describes the synthesis of ψdC and the synthesis of oligonucleotides and triphosphates containing it. The pure amidite *unit 13* can be prepared from thymidine (T) with a yield of 0.99% over 10 steps at a scale of ∼0.3 mmol. Using a 0.06 M solution of **13** in CH₃CN, oligo‐deoxynucleotides can be synthesized at a scale of 1.0 µmol using the conventional phosphoramidite method. Coupling is performed for 10 min using a 1H‐tetrazole solution (0.25 M) in acetonitrile as the activator, and capping requires the use of phenoxyacetic anhydride (Pac₂O) and pyridine in THF. The synthesized oligo‐deoxynucleotides are purified by RP‐HPLC, and the resulting oligonucleotides are identified by MALDI‐TOF MS measurements. The synthesis of the triphosphate of ψdC can also be accomplished from thymidine (T) at a scale of ∼0.3 mmol with a yield of 0.15% over 10 steps.

### Time Considerations

The synthesis of the amidite unit of the ψdC derivative (**13**) and its triphosphate (**16**) can be accomplished using thymidine with basic synthetic skills, each taking 3 weeks. Additionally, the synthesis of oligonucleotides incorporating the amidite unit requires 2 days, including DNA synthesis, purification, and confirmation.

### Author Contributions

**Ryo Miyahara**: Data curation; formal analysis; methodology; writing—original draft. **Yosuke Taniguchi**: Funding acquisition; supervision; validation; writing—review and editing.

### Conflict of Interest

The authors declare no conflict of interest.

## Data Availability

The data that support the findings of this study are available from the corresponding author upon reasonable request.
